# Genetic Variation in *ATP5O* Is Associated with Skeletal Muscle *ATP50* mRNA Expression and Glucose Uptake in Young Twins

**DOI:** 10.1371/journal.pone.0004793

**Published:** 2009-03-10

**Authors:** Tina Rönn, Pernille Poulsen, Tiinamaija Tuomi, Bo Isomaa, Leif Groop, Allan Vaag, Charlotte Ling

**Affiliations:** 1 Department of Clinical Sciences, Diabetes and Endocrinology Research Unit, CRC Malmö University Hospital, Lund University, Malmö, Sweden; 2 Steno Diabetes Center, Gentofte, Denmark; 3 Department of Medicine, University of Helsinki, Helsinki, Finland; 4 Department of Medicine, Helsinki University Central Hospital, Helsinki, Finland; 5 Genetic Institute, Folkhälsan Research Center, Helsinki, Finland; 6 Malmska Municipal Health Center and Hospital, Jakobstad, Finland; Istituto Dermopatico dell'Immacolata, Italy

## Abstract

**Background:**

Impaired oxidative capacity of skeletal muscle mitochondria contribute to insulin resistance and type 2 diabetes (T2D). Furthermore, mRNA expression of genes involved in oxidative phosphorylation, including *ATP5O*, is reduced in skeletal muscle from T2D patients. Our aims were to investigate mechanisms regulating *ATP5O* expression in skeletal muscle and association with glucose metabolism, and the relationship between *ATP5O* single nucleotide polymorphisms (SNPs) and risk of T2D.

**Methodology/Principal Findings:**

*ATP5O* mRNA expression was analyzed in skeletal muscle from young (*n* = 86) and elderly (*n* = 68) non-diabetic twins before and after a hyperinsulinemic euglycemic clamp. 11 SNPs from the *ATP5O* locus were genotyped in the twins and a T2D case-control cohort (*n* = 1466). DNA methylation of the *ATP5O* promoter was analyzed in twins (*n* = 22) using bisulfite sequencing. The mRNA level of *ATP5O* in skeletal muscle was reduced in elderly compared with young twins, both during basal and insulin-stimulated conditions (*p*<0.0005). The degree of DNA methylation around the transcription start of *ATP5O* was <1% in both young and elderly twins and not associated with mRNA expression (*p* = 0.32). The mRNA level of *ATP5O* in skeletal muscle was positively related to insulin-stimulated glucose uptake (regression coefficient = 6.6; *p* = 0.02). Furthermore, two SNPs were associated with both *ATP5O* mRNA expression (rs12482697: T/T versus T/G; *p* = 0.02 and rs11088262: A/A versus A/G; *p* = 0.004) and glucose uptake (rs11088262: A/A versus A/G; *p* = 0.002 and rs12482697: T/T versus T/G; *p* = 0.005) in the young twins. However, we could not detect any genetic association with T2D.

**Conclusions/Significance:**

Genetic variation and age are associated with skeletal muscle *ATP5O* mRNA expression and glucose disposal rate, suggesting that combinations of genetic and non-genetic factors may cause the reduced expression of *ATP5O* in T2D muscle. These findings propose a role for *ATP5O*, in cooperation with other OXPHOS genes, in the regulation of *in vivo* glucose metabolism.

## Introduction

Insulin resistance in skeletal muscle increases with age and during the course of type 2 diabetes mellitus (T2D). Reduced expression of genes from the respiratory chain in the mitochondria may cause impaired oxidative capacity in skeletal muscle which, in turn, has been suggested to contribute to insulin resistance [1]. Indeed, a number of studies have found reduced levels of genes involved in oxidative phosphorylation (OXPHOS) in parallel with increased insulin resistance in skeletal muscle from patients with T2D [2]–[5] and from elderly subjects [6]–[8]. Moreover, recent studies from our group showed positive associations between gene expression of the respiratory chain components *NDUFB6* and *COX7A1*, respectively, and insulin stimulated glucose uptake *in vivo* [7], [8]. Interestingly, a mRNA expression profile performed in our laboratory showed that *ATP5O* was the most significantly reduced OXPHOS gene in skeletal muscle from patients with T2D compared with healthy control subjects (*p* = 0.0027) [3]. However, the cause of this reduction in *ATP5O* remains unknown.

Oxidative phosphorylation is a process where electrons are passed from NADH and FADH_2_ along a series of carrier molecules, protons are pumped across the inner mitochondrial membrane to produce a proton gradient and in a final step ATP is produced from ADP and phosphate [9]. This process occurs in the respiratory chain, which consists of five multiprotein complexes. ATP5O is a nuclear encoded subunit of complex V of the respiratory chain i.e. ATP synthase. It is located in the stalk of the ATP synthase complex where it appears to connect the catalytic core (F_1_ subunit) and the membrane proton channel (F_0_ subunit), thereby influencing transmission of conformational changes and proton conductance [10].

The aims of the present study were to investigate 1) the mechanisms regulating *ATP5O* expression in skeletal muscle and 2) if single nucleotide polymorphisms (SNPs) in *ATP5O* are associated with increased risk of T2D.

## Materials and Methods

### Participants

#### Twins

Individuals were identified through The Danish Twin Register [11] and their clinical characteristics are described in Table 1. Selection criteria for the young and elderly twins, all non-diabetic, have been previously described [6], [11], [12].

**Table 1:** Clinical characteristics of participants.

	Young Twins	Elderly Twins	Botnia T2D Cases	Botnia Controls
*n* (male/female)	110 (60/50)	86 (38/48)	751 (399/352)	709 (344/365)
*n* (MZ/DZ)	110 (66/44)	86 (42/44)		
Age (years)	28.0±1.9	62.4±2.0*	54.5±9.4	53.5±11.5
BMI (kg/m^2^)	24.1±3.1	26.1±4.4*	28.9±4.8	25.9±3.8*
Glucose uptake (mg·kg LBM^−1^·min^−1^)	11.7±3.2	9.9±3.3*		
Fasting plasma glucose (mmol/l)			9.1±3.2	5.3±0.5*

Data are expressed as means±SD. DZ, dizygotic; MZ, monozygotic; LBM, lean body mass.

* *p*<0.05.

#### The Botnia study

This is a family based study established in 1990 aiming at the identification of T2D susceptibility genes [13]. 1461 unrelated individuals from the Botnia study were included in the present study, 751 of whom have been diagnosed with T2D >35 years of age and 709 non-diabetic controls without first degree relatives with T2D (Table 1). Subjects were classified into different stages of glucose tolerance based on an oral glucose tolerance test (OGTT). Individuals with genetically verified Maturity Onset Diabetes of the Young (MODY) or GAD antibody-positive patients were excluded from the study.

All studies were approved by the regional Ethics Committees and conducted according to the Helsinki Declaration. Written consent was obtained from all participants apart from a subset that was enrolled in the Botnia study in the early 90's when only verbal informed consent was obtained.

### Clinical examination

Each twin pair simultaneously underwent a 2-day clinical examination. The subjects were instructed to restrain from strenuous physical activity for 24 h and to fast overnight (10–12 h) before examination. Anthropometric measures (weight, height, BMI and waist to hip ratio) were performed as previously described [14]. Body composition (lean body mass [LBM] and fat mass) was determined by DEXA scanning. The subjects underwent a 2-hour hyperinsulinemic euglycemic clamp (40 mU m^−2^ min^−1^) combined with indirect calorimetry during the basal and insulin-stimulated steady state periods as previously described [14]. Metabolic rates (e.g. glucose disposal rate) were calculated as previously reported and expressed as mg·(kg LBM)^−1^·min^−1^ [15]. Plasma glucose and insulin concentrations were analyzed as previously described [11].

### Analysis of ATP5O mRNA levels in skeletal muscle

Muscle biopsies were obtained from the vastus lateralis muscle from the twins during both basal and insulin-stimulated states. Total RNA was extracted using Tri Reagent kit (Sigma-Aldrich, St. Louise, MO, USA) and cDNA was synthesized using Superscript II RNase H^−^ Reverse Transcriptase and random hexamer primers (Invitrogen, Carlsbad, CA, USA). *ATP5O* mRNA levels were quantified using TaqMan Real-Time PCR with an ABI 7900 system (Applied Biosystems, Foster City, CA, USA) using probe and primer pair specific for *ATP5O* covering exon boundary 4–5 (Assays-on-demands, Hs00426889_m1, Applied Biosystems). For the probe/primer set a standard curve was generated that was confirmed to increase linearly with increasing amounts of cDNA. Each sample was run in duplicates and the transcript quantity was normalized to the mRNA level of cyclophilin A (4326316E, Applied Biosystems), which expression was tested to not be affected by age.

### Genotyping

**Figure 1:**
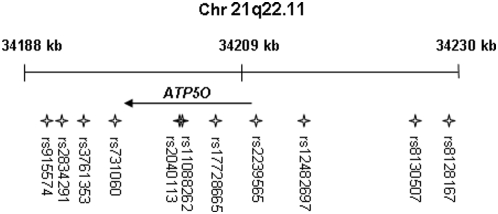
Chromosomal position of *ATP5O* and location of analyzed SNPs.

Genomic DNA was extracted from blood using conventional methods. Genotype data covering the *ATP5O* locus (chr21:34230028–34187627, 20 kb upstream and 10 kb downstream of the *ATP5O* gene transcript, **Fig. 1**) was downloaded from the International HapMap project (last accessed March 2008) [16] for the CEPH (Utah residents with ancestry from northern and western Europe) population. 33 SNPs with minor allele frequency >1% were found. Linkage disequilibrium (LD) structure between these SNPs was analyzed using Haploview [17] and 11 tag SNPs [18] were selected such that all alleles are correlated at an r^2^>0.9 (**Fig. 1**). These SNPs were genotyped using iPLEX™ assays and matrixassisted laser desorption/ionization time-of-flight (MALDI-TOF) mass spectrometry on the MassARRAY® system (Sequenom, San Diego, CA, USA) in the Swegene laboratory in Malmö. 3 SNPs failed this method and were analyzed by allelic discrimination using TaqMan® SNP Genotyping Assays on the ABI7900 (Applied Biosystems). Primer sequences and additional genotyping details are available from the authors.

### DNA Methylation

Genomic DNA was isolated from muscle biopsies at the same time as RNA using the Tri Reagent kit according to the manufacturer's instructions (Sigma-Aldrich). DNA bisulfite modification was accomplished on DNA from 11 young and 11 elderly twins using the EZ DNA methylation kit (Zymo Research, Orange, CA, USA). Bisulfite modified DNA was amplified with primers designed using the program MethPrimer [19], forward primer: 5′-TTGGTTAGTAATAATGATTTTATTTTTAGA -3′ and reverse primer 5′- CTAACTCTCAAAACCACCTTTCTCT -3′. A second, semi-nested PCR was performed using the forward primer 5′- GAGGGTTTAGTTTTTATTATTGTTAAGAAT -3′ (same reverse). The resulting amplicon is 249 basepairs long and includes 19 possible DNA methylation (CpG) sites. This region, which is located within a CpG island, surrounds the *ATP5O* transcription start site (TSS) including the beginning of exon 1 (−93 to +156 in relation to TSS). The PCR products were cloned into plasmid vectors (pCR®4-TOPO®, Invitrogen), further *E. coli* were transformed and DNA from 10 colonies of each individual muscle sample were isolated (QIAprep 8 Miniprep Kit, Qiagen Inc., Valencia, CA, USA). The individual clones were sequenced and the number of methylated sites was determined using BiQ Analyzer [20]. The sequences from all 22 individuals comprised of data from 8–10 clones. The proportion of methylation for each individual was calculated by dividing the total number of methylated sites in all clones by the total number of possible methylation sites.

### Statistical methods

#### Generalized estimating equations

Conventional tests of differences between variable (y) means are not valid for twin data due to the strong intrapair correlation. Generalized estimating equations (GEE) methodology (y = α+βx) was used to correct for this dependence and provide valid standard errors for the β-coefficients [21], [22]. All observations were used for estimation of the β-coefficient, whereas the variance of β was calculated using each twin pair as one cluster.

#### Linear regression

To identify factors independently associated with the response variable, we used backward-elimination multivariate regression analysis with *p*>0.05 as the defining criteria for exclusion of model terms. GEE methodology was used to obtain valid tests.

#### Biometric modeling

Intra-class correlations, a method to measure resemblance within twin pairs [23], were calculated for *ATP5O* mRNA expression in monozygotic (MZ) and dizygotic (DZ) twin pairs. The degree of genetic and environmental influence of a phenotypic variable can be estimated using biometric modeling as previously described [24]. The models tested included the following parameters: genetic variance due to additive genetic effects (V_A_) or dominant genetic effects (V_D_) and environmental variance due to an individual environment not shared with co-twin (V_E_) or a common environment shared among co-twins (V_C_). Heritability gives the proportion of the total variation (V_T_) of a trait attributable to additive genetic variation (V_A_). We used the MX software package, a programme for linear structural equation modeling, to estimate the variance components and to compare the different models. Biometric modeling was conducted separately in the two age groups. The fit of each model was assessed by maximum-likelihood methods and resulted in a χ^2^ goodness of fit index and probability value that tested the agreement between the observed and the predicted statistics. A small goodness of fit χ^2^ value, a high *p*-value and a low AIC (Akaike's information criterion), which equals the χ^2^ value minus 2 times the degree of freedom, indicates good correspondence and were used in comparisons of each model leading to a best fitting model.

#### Association between genetic variation in the *ATP5O* gene and type 2 diabetes

The Genetic Power Calculator [25] was used to calculate power for detection of association between SNPs in the *ATP5O* gene region and T2D. With a minor allele frequency of 5%, a T2D frequency of 6% and a relative risk of 1.3 at α = 0.05 we have 38% power using a dominant model in the Botnia case control study. Association of individual SNPs with T2D was determined by logistic regression adjusted for BMI, sex and age-at-onset (cases) or age-at-visit (controls), assuming an additive model. Hardy Weinberg *p*-value, LD and haplotype block structure were calculated using Haploview [17].

#### DNA methylation

Power to detect differences in DNA methylation in our study was calculated to 53.8% (α = 0.05) using DSS research statistical power calculator (www.dssresearch.com; Fort Worth, TX, USA).

## Results

### Age is associated with reduced skeletal muscle ATP5O mRNA expression levels

**Figure 2:**
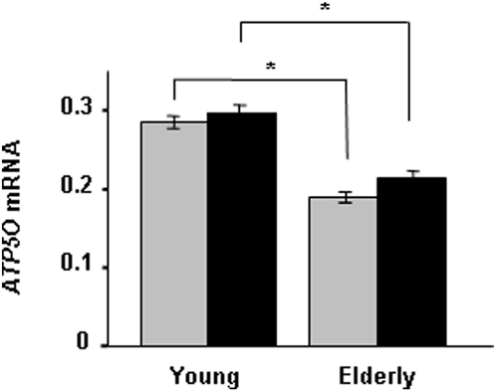
Age associates with *ATP5O* mRNA expression in skeletal muscle from young (*n* = 86) and elderly (*n* = 69) twins. Presented values are ratios between the mRNA expression of *ATP5O* and the endogenous control cyclophilin A, which was calculated for each sample and expressed as mean±SEM. Grey bars, before; and black bars, after a hyperinsulinemic euglycemic clamp. *p<0.05.

The influence of age on *ATP5O* mRNA expression was investigated in skeletal muscle biopsies taken from young and elderly twins, before and during a hyperinsulinemic euglycemic clamp. The mRNA level of *ATP5O* was reduced in elderly compared with young twins, both at the basal (0.19±0.007 versus 0.28±0.008; *p*<0.0005) and insulin-stimulated (0.21±0.009 versus 0.30±0.01; *p*<0.0005) state (**Fig. 2**).

### Heritability of skeletal muscle ATP5O mRNA expression

The degree of genetic and environmental influence on skeletal muscle *ATP5O* expression was estimated in young and elderly twins by means of intra-class correlations and biometric modeling. In the young twins, the intra-class correlation for skeletal muscle *ATP5O* expression was higher in MZ (0.77) as compared to DZ (0.34) with a heritability of 77% (**Table 2**). In contrast, intra-class correlations for skeletal muscle *ATP5O* expression were similar in elderly MZ and DZ twins, and explained by a combination of shared (41%) and unique (59%) environmental factors (**Table 2**).

**Table 2:** Heritability of *ATP5O* mRNA expression in skeletal muscle from 86 young and 69 elderly twins during clamp.

Best fitting biometric models		Components of variance			
	Model	Additive genetic a^2^	Dominant genetic d^2^	Common environment c^2^	Unique environment e^2^
Young	AE	0.77 (0.55–0.88)	-	-	0.23 (0.12–0.45)
Elderly	CE	-	-	0.41 (0.09–0.065)	0.59 (0.35–0.91)
Intra-class correlations		Young MZ	Young DZ	Elderly MZ	Elderly DZ
*ATP5O* clamp		0.77 (0.56–0.88)	0.34 (0.18–0.58)	0.50 (0.02–0.79)	0.55 (0.16–0.79)

Biometric modeling data are presented as proportion of total variance (95% CI). χ^2^ = 0.00, *p* = 1.00 and Akaike's information criteria = −2.00 for the goodness of fit tests for *ATP5O* expression during clamp in both young and elderly twins.

Correlation coefficients (95% CI) are presented for the intra-class correlation of *ATP5O* mRNA.

AE, model of additive genetic and specific environment variance; CE, model of shared and specific environment variance; DZ, dizygotic; MZ, monozygotic.

### Genetic variation is associated with ATP5O mRNA expression in muscle of young twins

**Figure 3:**
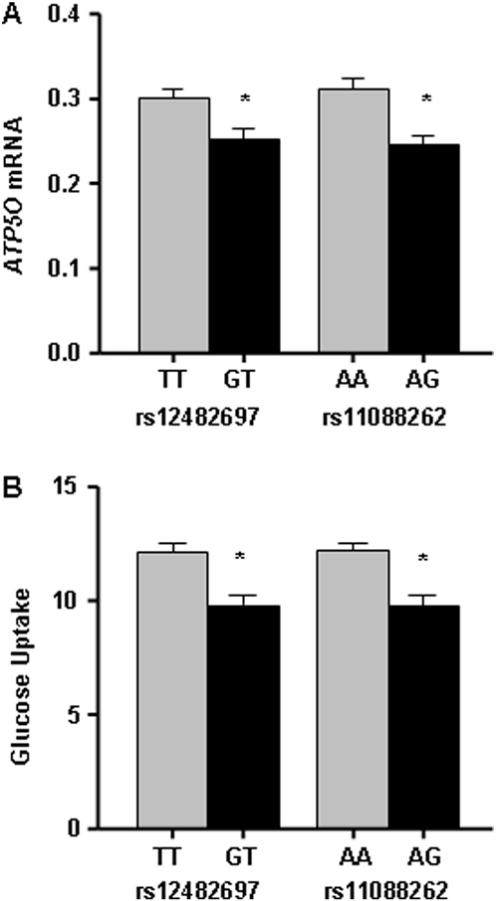
Two ATP5O SNPs are associated with both mRNA expression during clamp and insulin stimulated glucose uptake in young twins. Effects of rs12482697 respective rs11088262 on the *ATP50* mRNA levels (a) and glucose uptake (mg·kg LBM^−1^·min^−1^, b). Data are expressed as mean±SEM. *p<0.05.

In order to evaluate the genetic factors influencing *ATP5O* expression in muscle of the young twins, 11 tag SNPs (**Fig. 1**) were genotyped and related to gene expression. Two polymorphisms, rs12482697 and rs11088262, influenced the expression of *ATP5O* in muscle of the young twins (rs12482697: T/T [*n* = 65] 0.30±0.010 versus T/G [*n* = 16] 0.25±0.012; *p* = 0.02 and rs11088262: A/A [*n* = 70] 0.31±0.012 versus A/G [*n* = 16] 0.25±0.011; *p* = 0.004) during the clamp (**Fig. 3A**). These two polymorphisms are likely to represent the same association, as they show strong LD (r^2^ = 0.96). rs12482697 is located 5 kb upstream from transcription start whereas rs11088262 is a missense polymorphism in exon 4. However, none of the other SNPs were associated with *ATP5O* expression in skeletal muscle (data not shown).

### DNA methylation in the ATP5O promoter

We also tested if DNA methylation around the transcription start of *ATP5O* may influence the mRNA expression in skeletal muscle. The degree of DNA methylation was low in both young (0.14±0.07%; *n* = 11) and elderly (0.73±0.33%; *n* = 11) twins and there was no significant difference between the two age-groups (*p* = 0.16). Of note, eight young and six elderly twins showed no methylation at all of this DNA sequence. Furthermore, we could not observe any significant correlation between *ATP5O* DNA methylation and mRNA expression (*r* = 0.24; *p* = 0.32).

### Additional factors associated with ATP5O expression in skeletal muscle

To examine if additional factors are associated with *ATP50* expression in muscle a multivariate regression analysis was performed including the following parameters as explanatory variables: basal and insulin-stimulated skeletal muscle *PGC-1α* and *PGC-1β* mRNA expression, birth weight, age, sex, BMI and zygosity (**Table S1**). The final model was reached using backward selection regression. *ATP5O* expression was positively related to insulin-stimulated *PGC-1α* expression (regression coefficient = 0.082, *p* = 0.0001) and inversely related to age (regression coefficient = −0.001, *p* = 0.0001) and female sex (regression coefficient = −0.043, *p* = 0.001).

### Impact of ATP5O on in vivo glucose uptake

Oxidative capacity has been suggested to influence insulin sensitivity. We therefore performed a regression analysis to test if *ATP5O* expression in skeletal muscle is related to *in vivo* insulin stimulated glucose uptake in young and elderly twins (*n* = 155). The following variables were included in the regression model: *ATP5O* mRNA expression, zygosity, birth weight, age, sex and BMI. Indeed, the mRNA level of *ATP5O* was positively related to glucose uptake (regression coefficient = 6.6; *p* = 0.02). Furthermore, since rs11088262 and rs12482697 influenced the expression level of *ATP5O* in skeletal muscle of the young twins, we tested if these polymorphisms also were associated with glucose uptake. Both rs11088262 and rs12482697 showed association with insulin-stimulated glucose uptake in the young twins (rs11088262: A/A [*n* = 85] 12.2±0.4 versus A/G [*n* = 18] 9.8±0.5; *p* = 0.002 and rs12482697: T/T [*n* = 79] 12.1±0.36 versus T/G [*n* = 18] 9.8±0.6; *p* = 0.005, **Fig. 3B**), but not in elderly twins.

### ATP5O polymorphisms and risk of T2D

11 common variants in the *ATP5O* gene region were genotyped in the Botnia case-control cohort in order to assess association to T2D. None of the SNPs showed significant association to T2D (**Table S2**).

## Discussion

The key findings of the present study were that genetic variation in the *ATP5O* gene region is associated with mRNA expression in skeletal muscle and glucose uptake in young twins. Furthermore, aging proves to have a negative effect on *ATP5O* mRNA expression, which is in line with findings for other OXPHOS genes [7], [8]. Also the positive correlation between the transcriptional co-activator *PGC-1α* and OXPHOS genes previously shown [3] could be confirmed in our study.

*ATP5O* was selected for analysis in this study because it was previously shown to be the most strongly downregulated OXPHOS gene in diabetic muscle [3]. Furthermore, it encodes a component of ATP synthase, the complex in the respiratory chain responsible for the final step of ATP production. Including twins in the study design gives the opportunity to estimate the relative influence of genetic versus environmental components. Interestingly, the genetic influence on *ATP5O* mRNA expression estimated by both biometric modeling and intra-class correlation was not seen in the elderly subjects, which may suggest an accumulation of environmental factors with advancing age, which masks the genetic component contributing to the *ATP5O* expression demonstrated in young individuals.

Age is positively associated with insulin resistance and the prevalence of T2D. Furthermore, a correlation between insulin sensitivity and the oxidative capacity of skeletal muscle has been reported [26]. The glucose disposal rate calculated from a hyperinsulinemic euglycemic clamp is a reliable measure of insulin sensitivity. In the present study, the *ATP5O* mRNA expression was reduced by one third in elderly compared to young twins, with a concomitant decrease in glucose uptake. This is in line with other reports suggesting that age has a strong influence on mitochondrial OXPHOS activity or OXPHOS gene expression as well as on insulin-stimulated glucose uptake in skeletal muscle [7], [27]–[29]. To understand the mechanisms responsible for this decline it would be useful to relate mRNA expression to mitochondrial content and respiratory chain activity in forthcoming studies.

The association between two genetic variants (rs11088262 and rs12482697) and glucose uptake was only seen in young but not in elderly twins. This was in agreement with the heritability estimates, where the heritability of *ATP5O* mRNA expression was estimated to 77% in the young twins, but only environmental factors were found to influence *ATP5O* expression in the elderly twins. However, we could not find any genetic association between *ATP5O* SNPs and T2D in our case control cohort. T2D is a result of several pathophysiological mechanisms, e.g. impaired insulin secretion and insulin resistance in the liver and peripheral tissues. Here we find in non-diabetic twins an association between SNPs and glucose uptake, whereas the T2D cases are characterized by a concert of different players where perhaps an insulin secretional defect may play the lead part and mask an association with insulin resistance. Also, this could be due to low power as we only had 38% power to detect an association using this design and recent genome-wide association studies suggest that very large cohorts are needed to detect modest association commonly seen for T2D [30]. To partially compensate for low power we also analyzed data from a genome-wide association scan performed by our laboratory [31] and a T2D meta-analysis [32] for this region of the *ATP5O* locus (chr21:34230028–34187627). A total number of 32 SNPs (including imputed data) were analyzed but none showed evidence for association with T2D.

In the promoter region of some but not all OXPHOS genes, increased DNA methylation in elderly subjects has been reported [7], [8]. For *PGC-1α*, one of the master regulators of OXPHOS genes, there was no difference in the level of DNA methylation in skeletal muscle of young and elderly twins [7], but an increase was found in human pancreatic islets from T2D compared to islets from healthy organ donors [33]. With this background we wanted to investigate the level of DNA methylation in the *ATP5O* promoter region, but hardly any DNA methylation could be detected and there was no difference between the two age groups.

In conclusion, both genetic variation and age were associated with skeletal muscle *ATP5O* mRNA expression and glucose disposal rate, suggesting that combinations of genetic and non-genetic factors may cause the reduced expression of *ATP5O* in T2D muscle. These findings propose a role for *ATP5O*, in cooperation with other OXPHOS genes, in the regulation of *in vivo* glucose metabolism.

## Supporting Information

Table S1 (0.03 MB DOC)

Table S2 (0.04 MB DOC)
