# Supplementary material for: Genetic Variation in ATP5O Is Associated with Skeletal Muscle ATP50 mRNA Expression and Glucose Uptake in Young Twins
Source: PLoS One. 2009 Mar 10;4(3):e4793. doi: 10.1371/journal.pone.0004793 (PMC2651471; doi:10.1371/journal.pone.0004793)
Supplement: Table S1 — (0.03 MB DOC) [file pone.0004793.s001.doc]

**Table S1** Factors influencing skeletal muscle *ATP5O* mRNA expression in 86 young and 69 elderly twins during clamp

| **ATP5O** | **Regression coefficients** | ***p-*value** |
| --- | --- | --- |
| *PGC-1α* clamp | 0.082 | 0.0001 |
| Age (young vs. elderly) | -0.001 | 0.0001 |
| Sex (male vs. female) | -0.043 | 0.001 |

By regression analysis we tested whether the following factors: basal and insulin-stimulated skeletal muscle *PGC-1α* and *PGC-1β* mRNA expression, birth weight, zygoti, age (young coded 0 and elderly coded 1), sex (male coded 0 and female coded 1) and BMI influence insulin-stimulated mRNA expression of *ATP5O* in skeletal muscle. All final models have *p* < 0.05
