# Supplementary material for: Genetic Variation in ATP5O Is Associated with Skeletal Muscle ATP50 mRNA Expression and Glucose Uptake in Young Twins
Source: PLoS One. 2009 Mar 10;4(3):e4793. doi: 10.1371/journal.pone.0004793 (PMC2651471; doi:10.1371/journal.pone.0004793)
Supplement: Table S2 — (0.04 MB DOC) [file pone.0004793.s002.doc]

**Table S2** Association between SNPs in the *ATP5O* gene locus and type 2 diabetes in the Botnia case-control cohort

| SNP ID | Alleles major/minor | HWP  case/control | MAF  case/control | Genotype frequency (case/control) aa aA AA | | | Odds ratio (95% CI) | *p*-value | power |
| --- | --- | --- | --- | --- | --- | --- | --- | --- | --- |
| rs915574 | C/T | 0.63/0.06 | 0.362/0.366 | 0.402/0.385 | 0.472/0.494 | 0.126/0.121 | 1.00 (0.85-1.19) | 0.97 | 62% |
| rs2834291 | C/T | 0.71/0.88 | 0.394/0.404 | 0.357/0.371 | 0.476/0.470 | 0.167/0.162 | 0.97 (0.83-1.14) | 0.71 | 58% |
| rs3761353 | A/G | 0.78/1.00 | 0.198/0.200 | 0.645/0.645 | 0.314/0.315 | 0.041/0.040 | 1.01 (0.82-1.23) | 0.96 | 69% |
| rs731060 | T/C | 0.52/0.002 | 0.306/0.292 | 0.487/0.527 | 0.414/0.362 | 0.099/0.111 | 1.08 (0.91-1.27) | 0.37 | 66% |
| rs2040113 | G/A | 0.46/0.24 | 0.471/0.466 | 0.287/0.275 | 0.484/0.516 | 0.229/0.208 | 1.00 (0.86-1.18) | 0.95 | 47% |
| rs11088262 | A/G | 0.74/0.65 | 0.101/0.114 | 0.807/0.785 | 0.185/0.204 | 0.008/0.011 | 0.81 (0.62-1.06) | 0.13 | 57% |
| rs17728665 | A/G | 1.00/1.0 | 0.031/0.028 | 0.939/0.943 | 0.059/0.057 | 0.001/0.000 | 0.93 (0.58-1.49) | 0.75 | 27% |
| rs2239565 | C/T | 0.61/0.61 | 0.072/0.071 | 0.863/0.861 | 0.130/0.136 | 0.007/0.003 | 1.02 (0.85-1.38) | 0.91 | 48% |
| rs12482697 | T/G | 1.00/0.58 | 0.100/0.116 | 0.809/0.780 | 0.182/0.210 | 0.009/0.011 | 0.78 (0.60-1.02) | 0.07 | 57% |
| rs8130507 | C/T | 0.30/0.81 | 0.420/0.416 | 0.347/0.343 | 0.467/0.477 | 0.186/0.180 | 1.01 (0.86-1.18) | 0.92 | 55% |
| rs8128167 | T/C | 0.48/0.56 | 0.399/0.391 | 0.354/0.382 | 0.494/0.461 | 0.152/0.158 | 1.06 (0.90-1.25) | 0.48 | 57% |

HWP: Hardy Weinberg *p*-value, MAF: minor allele frequency, aa: reference genotype, Odds ratio: calculated for additive model using logistic regression adjusted for age, sex and BMI. Shown *p*-values have not been corrected for multiple testing. Power for detection of association was calculated using α=0.05, assuming a T2D frequency of 6% and a relative risk of 1.3
